# Intraoperative Cholangiogram Facilitates Single-Session Laparoscopic Cholecystectomy and Intraoperative Endoscopic Retrograde Cholangiopancreatography: Case Reports and Review of the Literature

**DOI:** 10.7759/cureus.71444

**Published:** 2024-10-14

**Authors:** Colin R Tang-Whitmore, Bridget S Dillon, Taylor Benedict, Matthew H Wheelwright, Nabeel Azeem, Stuart Amateau, James V Harmon

**Affiliations:** 1 Department of Medicine, University of Minnesota Medical School, Minneapolis, USA; 2 Department of Diagnostic Radiology, University of Miami Miller School of Medicine, Miami, USA; 3 Department of Surgery, University of Minnesota Medical School, Minneapolis, USA; 4 Department of Gastroenterology, University of Minnesota Medical School, Minneapolis, USA

**Keywords:** case report, choledocholithiasis, ercp, ioc, lc

## Abstract

Choledocholithiasis is reported in up to 20% of patients undergoing cholecystectomy. It is recommended to remove common bile duct gallstones due to the risk of complications. A common approach to the management of choledocholithiasis is to combine laparoscopic cholecystectomy (LC) with endoscopic retrograde cholangiopancreatography (ERCP), which is typically completed during two separate sessions. The use of intraoperative cholangiogram (IOC) to facilitate combined LC and intraoperative endoscopic retrograde cholangiopancreatography (iERCP) may reduce overall operative time and hospital length of stay. We report two patients who underwent LC and iERCP under the same anesthetic administration for the treatment of choledocholithiasis. A large data set on the role of IOC in completing both LC and iERCP under a single anesthetic administration was analyzed. These two patients illustrate the utility of IOC in facilitating single-session LC/iERCP for the treatment of choledocholithiasis and our retrospective analysis highlights the effectiveness of IOC.

## Introduction

The benefits of single-session laparoscopic cholecystectomy (LC) and intraoperative endoscopic retrograde cholangiopancreatography (iERCP) to manage choledocholithiasis have been demonstrated in several studies [1-6]. The intraoperative cholangiogram (IOC) procedure can be used to identify patients likely to benefit from the combined LC/iERCP approach. The combined LC/iERCP procedure reduces exposure to general anesthesia and is associated with a shorter length of hospital stay and decreased costs [2]. We report two patients in whom IOC confirmed choledocholithiasis to facilitate the completion of LC and iERCP under a single anesthetic. To assess the effectiveness of IOC in general we retrospectively analyzed iERCP findings in 12 sequential patients undergoing single-session combined procedures who completed successful IOC at our center.

## Case presentation

Case 1

A 74-year-old man presented to the Emergency Department (ED) with intermittent right upper quadrant (RUQ) pain, abdominal bloating, and nausea. The patient’s previous medical history included gastroesophageal reflux disease and hyperlipidemia. Upon examination, the patient had normal vital signs and was afebrile. The abdomen was soft and non-distended with localized RUQ tenderness, and a positive Murphy sign was elicited. The patient’s peripheral white blood cell count was 4.3 (4.0-11.0) 10^3^/mL and the hemoglobin was 12.9 (normal 11.7-15.7) g/dL. The aspartate transaminase (AST) was 84 (normal 0-45) U/L; alanine transaminase (ALT) was 130 (normal 0-50) U/L; alkaline phosphatase (ALP) was 192 (normal 40-150) U/L; total bilirubin was 3.6 (normal 0.2-1.3) mg/dL; lipase was 116 (normal 73-393) g/dL; and serum albumin was 2.8 (normal 3.4-5.0) g/dL.

**Figure 1:**
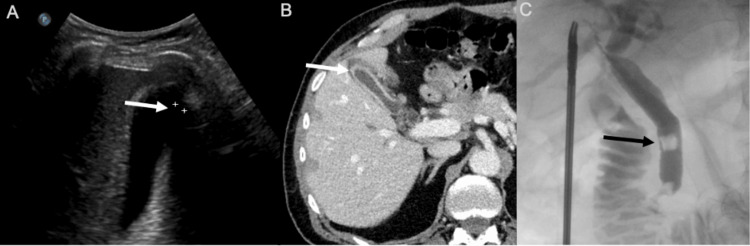
(A) Ultrasound examination of the RUQ revealed mild thickening of the gallbladder wall without cholelithiasis and dilation of the CBD to 9.3 mm without choledocholithiasis. The full length of the CBD could not be visualized using ultrasonography. (B) Contrast-enhanced CT scan of the abdomen showed gallbladder wall thickening and possible sludge or non-calcified stones in the distal portion of the CBD. (C) IOC revealed multiple gallstones in the CBD RUQ: right upper quadrant; CBD: common bile duct; IOC: intraoperative cholangiogram

A RUQ ultrasound exam revealed a thickened gallbladder wall and a 9.3 mm dilated common bile duct (CBD) (Figure 1A). However, no stones were identified in the gallbladder or the CBD. A contrast-enhanced abdominal CT scan revealed gallbladder wall thickening and possible sludge or non-calcified stones in the distal portion of the CBD (Figure 1B).

The patient was scheduled for LC, IOC, and possible iERCP. The gallbladder demonstrated findings of chronic inflammation. The IOC revealed multiple gallstones in the CBD (Figure 1C). Based on the IOC findings, iERCP was performed after LC completion. An 8-mm biliary sphincterotomy was performed, and multiple, large gallstones and biliary concretions were cleared during the iERCP using a 15-mm balloon catheter. Pathological examination confirmed chronic cholecystitis. The patient was discharged home on postoperative day two and is without complications at the two-year follow-up.

Case 2

A 74-year-old woman with known gallstones presented to the ED with nausea and severe abdominal pain following a fatty meal. The patient’s prior medical conditions included atrial fibrillation managed with warfarin, hyperlipidemia, hypertension, hypothyroidism, and obesity. Upon examination, the patient had normal vital signs and was afebrile. The abdomen was soft and non-tender, a Murphy sign could not be elicited. The patient’s white blood cell count was 7.8 (normal 4.0-11.0) 10^3^/mL and the hemoglobin was 12.9 (normal 11.7-15.7) g/dL. The AST was 72 (normal 0-45) U/L, ALT was 82 (normal 0-50) U/L, ALP was 122 (normal 40-150) U/L, total bilirubin was 0.6 (normal 0.2-1.3) mg/dL, lipase was < 50 (normal 73-393) g/dL, and serum albumin was 2.3 (normal 3.4-5.0) g/dL. The patient’s initial international normalized ratio (INR) was 3.4 (normal 0.85-1.15) and was reversed to 1.49 following administration of vitamin K.

**Figure 2:**
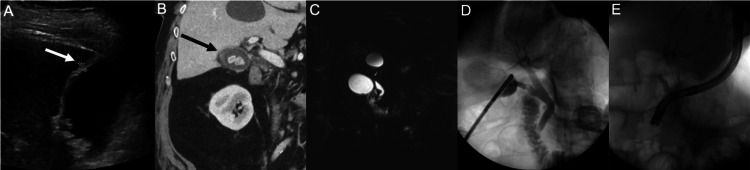
(A) Abdominal ultrasonography showing a slightly thickened gallbladder wall with gallstones (B) Contrast-enhanced abdominal computed tomography scan revealing cholelithiasis with thickening of the gallbladder wall (C) MRCP (D) IOC (E) Fluoroscopy MRCP: magnetic resonance cholangiopancreatography; IOC: intraoperative cholangiogram

A RUQ ultrasound exam demonstrated gallbladder wall thickening and dilation of the CBD to 13 mm (Figure 2A). A contrast-enhanced abdominal and pelvic CT scan demonstrated gallbladder wall thickening, scant pericholecystic fluid, and a dilated CBD (Figure 2B). Magnetic resonance cholangiopancreatography (MRCP) revealed dilation of the CBD to 10 mm and possible choledocholithiasis (Figure 2C).

The patient underwent LC, IOC, and iERCP. The gallbladder demonstrated findings of chronic inflammation. Although no stones were found in the CBD, IOC confirmed the presence of a gallstone within the distal cystic duct (Figure 2D). The cystic duct gallstone was manipulated into the CBD using an atraumatic laparoscopic grasper to facilitate removal by iERCP. The gallstone was confirmed to be within the CBD with a subsequent IOC (Figure 2E). Based on the IOC findings, iERCP was completed after the LC. iERCP revealed stone and sludge in the lower third of the CBD. A 9 mm biliary sphincterotomy was performed and a single gallstone and sludge were swept from the CBD using a 12 mm balloon catheter. A 10-Fr by 5 cm Sofflex plastic biliary stent was placed in the patient’s CBD (Figure 2E). Pathologic examination revealed chronic cholecystitis with cholelithiasis. The patient was discharged on postoperative day one and is without complications at the two-year follow-up.

## Discussion

Methods

We performed retrospective chart reviews of two patients who underwent IOC to facilitate combined LC and iERCP during a single anesthetic. Both patients had a high likelihood of choledocholithiasis based on their clinical history, examination findings, laboratory data, and imaging results. 

We also retrospectively analyzed the patient characteristics, lab findings, cost of care, and outcomes for a cohort of 33 patients who underwent combined LC/iERCP at our academic center. We then focused our analysis on 12 patients who completed a successful IOC during LC prior to iERCP during the same anesthetic.

Standard t-tests were used to assess continuous variables. The t-test function of Microsoft Excel (version 16.74: Microsoft Corporation, Redmond, Washington, USA) was used to perform t-tests. The t-tests were two-tailed and assumed equal group variances. P values less than 0.5 were considered statistically significant.

Results

The two patients reported undergoing combined LC, IOC, and iERCP. Successful IOCs demonstrated the presence of choledocholithiasis and iERCP procedures were successful in clearing the CBD under a single anesthetic. Both patients have had no complications associated with their procedures at two-year follow-up.

All 33 patients in the combined LC/iERCP cohort were at high risk of choledocholithiasis based on their presentation, laboratory findings, and imaging studies. Gallstones were cleared from the CBD in 85% (28/33) of patients. Sludge was cleared from the CBD in 42% (14/33) of patients. All patients had evidence of either gallstones or sludge in the CBD at the time of iERCP. In total, 15 patients from the combined procedure cohort had either completed or attempted IOC prior to iERCP. Successful IOCs were completed in 80% (12/15) of IOCs attempted. We focused our analysis on the findings related to the 12 patients who completed successful IOCs. The IOC was interpreted as positive in 58% (7/12) of patients. The IOC was interpreted as indeterminate in 33% (4/12) of patients. The IOC was interpreted as negative in 8% (1/12) of patients. Gallstones were cleared from the CBD in all patients with positive or indeterminate IOC. Sludge was cleared from the CBD of the single patient with a negative IOC. Gallstones were cleared from the CBD in 67% (2/3) of patients who had IOC that was attempted, but not completed. Sludge was cleared from the CBD in 33% (1/3) of patients who had IOC that was attempted, but not completed. Gallstones were cleared from the CBD in 83% (15/18) of patients who did not have IOC attempted. There were no complications associated with IOC completion in this patient cohort.

Patient characteristics, lab findings, and outcomes were compared between patients who underwent combined LC/iERCP without IOC and those who underwent combined LC/iERCP with IOC. No significant differences were identified between the two groups, except total operative time (Table 1). The total average operative time was 124±49 min in patients who did not undergo IOC and 164±61 min in those who did undergo IOC (p=0.044). The average total cost estimates for the patients in the LC/iERCP without an IOC cohort was $44,982±$28,771. The average total cost estimates for the patients in the LC/iERCP with IOC cohort was $38,761± $13,481. The average total cost estimates were not significantly different for the patients in these two cohorts (p=0.462).

**Table 1:** Characteristics of the patients who underwent single-session LC/iERCP separated by IOC *Postoperative fever with negative culture **n=14 LC: laparoscopic cholecystectomy; ERCP: endoscopic retrograde cholangiopancreatography; IOC: intraoperative cholangiography; CBD: common bile duct; SD: standard deviation

	Value	LC-›ERCP	LC-›IOC-›ERCP	P-value
		n=18	n=15	
Sex	Male/female	5/13	4/11	0.945
Age	Mean±SD	50.3±23.5	46.5±13.5	0.583
Number of CBD stones	0/1/2+	3/4/11	2/7/6	0.502
CBD stone size (mm)	Mean ± SD	5.5±3.2, n=10	6.3±2.5, n=9	0.524
CBD sludge	Present (%)	8 (44)	6 (40)	0.805
Operative duration (min)	Mean±SD	124±49	164±61	0.044
Anesthesia duration (min)	Mean±SD	216±61	242±68	0.265
Length of hospital stay (days)	Mean±SD	4.9±2.6	4.6±2.7	0.714
Adverse events	Present (%)	0 (0)	1 (7)*	-
Total cost of care (United States Dollars, unadjusted)	Mean±SD	44,982±28,771	38,761±13,481**	0.462
Alanine aminotransferase level (U/L)	Mean±SD	235.2±254.9	184.5±139.65	0.497
Alkaline phosphatase level (U/L)	Mean±SD	217.1±137.5	151.3±79.7	0.118
Aspartate aminotransferase level (U/L)	Mean±SD	138.7±129.2	92.4±80.7	0.239
Amylase level (U/L)	Mean±SD	20.0±33.3	17.5±32.2	0.831
Bilirubin level (mm/dL)	Mean±SD	2.1±1.5	1.2±1.0	0.057
International normalized ratio (INR)	Mean±SD	1.1±0.1	1.1±0.1	0.821
White blood cell count (10^9 ^cells/L)	Mean±SD	7.2±2.5	6.7±3.6	0.690

Discussion

Choledocholithiasis is reported in up to 20% of patients undergoing cholecystectomy; gallstones located in the CBD often pass spontaneously within six weeks [7]. However, it is generally recommended to remove CBD stones due to the risk of acute pancreatitis and ascending cholangitis. The presence of gallstones in the CBD is often identified incidentally; in one study, over half of the CBD stones were found through routine IOC [8]. If LC is completed prior to an ERCP the identification of choledocholithiasis using cholangiography may assist in planning interventions if stones are identified [9].

Performing preoperative ERCP in an endoscopy suite, followed by performing an LC in the operating room under separate anesthetic administrations, is a common clinical pathway for patients with choledocholithiasis. This approach requires two anesthetic sessions, delays definitive care, and extends the length of hospital stay. Performing single-session combined LC and iERCP procedures has been increasingly recognized in choledocholithiasis management guidelines. Thirty-three patients in our study group underwent combined LC and iERCP. IOCs were attempted in 15 of these 33 patients and successfully completed in 12 patients prior to ERCP. An analysis of these 12 patients demonstrated the effectiveness of the IOC as each of these patients immediately underwent iERCP. All patients with a positive or indeterminate IOC were found to have choledocholithiasis during iERCP. Other investigators report the specificity of IOC to range from 92%-99% [10,11]. Iranmanesh et al. compared 100 patients who were randomized to undergo LC with IOC versus the outcomes for patients who underwent ERCP followed by LC. Patients found to have CBD gallstones at the time of IOC underwent either iERCP or postoperative ERCP. Patients undergoing LC with IOC and iERCP or postoperative ERCP had a significantly shorter length of hospital stay (five versus eight days) than patients undergoing preoperative ERCP followed by LC (p<0.001). There was no significant difference in terms of mortality and morbidity rates between the patients in these two groups [12]. LC with IOC and iERCP was the approach taken for the two patients in our case reports, for all patients in our retrospective analysis, and for the majority of patients we care for at our medical center. ERCP expertise is readily accessible at our hospital due to the proximity of the ERCP ORs to our operating rooms, and we have conducted combined cholecystectomy and iERCP procedures for years [2]. Choledocholithiasis can also be managed through laparoscopic exploration of the CBD. Surgeons at many centers have the expertise to perform laparoscopic transcystic exploration of the CBD and report the ability to clear the CBD in up to 80% of patients. We endorse this approach and have published an analysis of national database outcomes demonstrating that laparoscopic CBD explorations provide an advantage to patients when surgical teams are experienced with this approach [4]. However, not all facilities have the experience necessary to perform laparoscopic CBD exploration. Our approach can be used to definitively manage choledocholithiasis while still reducing instances of preoperative ERCPs.

MRCP is a useful imaging modality for the identification of CBD stones. MRCP findings can trigger preoperative ERCP to clear the CBD prior to LC. We attempt to limit the use of preoperative ERCP due to the risks associated with unnecessary ERCPs including perforation of the CBD, retroperitoneal leak and abscess, and ERCP-induced pancreatitis. As we try to limit preoperative ERCPs, MRCP imaging is less frequently obtained at our center. Our approach opts for the use of an IOC between the LC and possible ERCP potentially performed during the same operative session. This approach requires fewer MRCP evaluations. We appreciate, however, that this approach is not applicable to all clinical scenarios. In patients with ascending cholangitis, ERCP should be performed first, and LC deferred until the patient has stabilized.

Although there are benefits to performing the IOC, there remains a concern regarding the time required to perform IOCs. Our retrospective analysis found that there was a statistically significant difference (p=0.004) in operative times between patients who underwent IOC (164±61 min) and those who did not (124±49 min). A review of the literature reveals that the IOC procedure adds approximately 12 minutes (range of two to 30 minutes) (Table 2). When iERCP is performed in combination with LC the overall operative and anesthesia times are reduced compared to performing pre or postoperative ERCP.

**Table 2:** Summary of a review of recent literature on additional time to complete IOC ^a^IOC utilizing Kumar clamp ^b^IOC utilizing Olsen clamp ^c^Weighted mean difference IOC:  intraoperative cholangiography

Publication	Number of patients	Mean IOC time	Median IOC time	IOC time range
Shallaly, et al. [13]	72	14 min	-	-
Wenner, et al. [14]	52	4 min	3 min	2-16 min
Ford, et al. [15]	1715	16 min	-	10-23 min
Buddingh, et al. [16]	28^a^/19^b^	11 min^a^/12 min^b^	-	-
Hope, et al. [17]	54	11 min	-	6-22 min
Giulea, et al. [18]	147	-	11 min	-
Kovács, et al. [19]	10,735	11 min^c^	-	-

At our academic center, surgical trainees actively participate in the operative care of our patients. Performing and interpreting IOCs is a critical aspect of surgical training. Surgical trainees may find the use of the Kumar clamp aids in performing successful IOCs. The Kumar clamp is applied across the infundibulum of the gallbladder, just above Hartmann’s pouch. The gallbladder is then punctured with the needle, and the biliary tree can be visualized radiographically. Experience interpreting IOC images is also important, and Kaldas et al. report significant improvement in surgical trainees’ ability to interpret IOCs after using an online curriculum [20]. 

## Conclusions

In conclusion, the two patients we present graphically illustrate IOC findings during single-session LC-iERCP for the treatment of choledocholithiasis. Our retrospective analysis of patients who underwent successful IOC highlights the effectiveness of IOC. Within health care systems where laparoscopic clearance of CBD stones is not routine, selective use of IOC to identify the presence of CBD stones during LC followed by iERCP offers reduced overall procedure time, length of hospital stay, and cost compared to either preoperative or postoperative ERCP.
